# Effects of Self-Assisted Manual Therapy Combined with a High-Intensity Walking Program on Musculoskeletal Pain, Functionality, and Posture in Older Adults: A Multicentre Randomized Controlled Trial

**DOI:** 10.3390/life15060844

**Published:** 2025-05-23

**Authors:** Gemma Victoria Espí-López, Laura Fuentes-Aparicio, Rocío Cogollos-de-la-Peña, Lucas Monzani, Elena Marques-Sule, Dagmar Pavlu, Anna Arnal-Gómez

**Affiliations:** 1 Department of Physiotherapy, Faculty of Physiotherapy, University of Valencia, Gascó Oliag St., 5., 46010 Valencia, Spain; gemma.espi@uv.es (G.V.E.-L.); rocioinmaculada.cogollos@universidadeuropea.es (R.C.-d.-l.-P.); elena.marques@uv.es (E.M.-S.); anna.arnal@uv.es (A.A.-G.); 2 Exercise Intervention for Health Research Group (EXINH-RG), Department of Physiotherapy, University of Valencia, 46010 Valencia, Spain; 3 Physiotherapy in Motion, Multispeciality Research Group (PTinMOTION), Department of Physiotherapy, University of Valencia, Gascó Oliag St., 5, 46010 Valencia, Spain; 4 Department of Physiotherapy, Faculty of Health Science, Universidad Europea de Valencia, Pg. de l’Albereda, 7, El Pla del Real, 46010 Valencia, Spain; 5 Ivey Business School, Western University, 1255 Western Rd., London, ON N6G 0N1, Canada; lmonzani@ivey.ca; 6 Faculty of Physical Education and Sport, Charles University, 162 52 Prague, Czech Republic; dagmar.pavlu@ftvs.cuni.cz

**Keywords:** aging, pain, functional capacity, physical therapy, physical activity

## Abstract

Background: Aging is associated with musculoskeletal pain and postural adaptations which may affect functionality. This study aims to analyse the effect of a combined protocol of self-assisted manual therapy and high-intensity walking on musculoskeletal back pain, functionality, and shoulder posture in older adults, and to establish the short- and medium-term effects of this program. Methods: A multicentre, double-blind, randomized trial was conducted on older adults. The sample was divided into two groups: the self-assisted manual therapy plus walking at high-intensity group (MTWG) and the Control Group (CG), with the latter undergoing supervised high-intensity walking only. Pain (Pressure Pain Threshold and Visual Analogue Scale), functional capacity (5-times sit-to-stand test), and change in thorax position (acromion position) were assessed at T0 (baseline), T1 (after 4-week intervention) and T2 (follow-up, 4 weeks after the end of the intervention). Results: A total of 95 older adults (41 in the MTWG and 54 in CG) completed the study and were analyzed. After isolating the effect of correlations among our primary outcomes, our analysis revealed statistically significant between-subject (*p* < 0.01), within-subject (*p* < 0.001) and between-within subject differences (*p* < 0.05) in Pressure Pain Threshold scores in favour of the MTWG. We also detected within-subjects (*p* < 0.001) and between-within subject differences (*p* < 0.05) in scores for the Visual Analogue Scale, in favour of the MTWG. These patterns of results remained stable at T2. The 5-times sit-to-stand test (*p* < 0.01) and the acromion position (*p* < 0.05) improved at T1 for the MTWG but not at T2. Conclusions: A combined protocol of self-assisted manual therapy and high-intensity walking, compared to high-intensity walking alone, improved musculoskeletal pain, functionality, and posture in older adults in the short term (over one month), with pain reduction maintained in the medium term (at the one-month follow-up).

## 1. Introduction

There is currently an increase in the number of older adults in the general population worldwide, especially in proportion to younger individuals, and it is estimated that between 2020 and 2030 the global percentage of people over 60 years of age will increase by 34% [1]. Therefore, global health care demands [2], considering social, economic, and biomedical perspectives, aim to achieve longer and healthier lives and to reduce the costs associated with this population [3].

Older adults can experience impairments during ageing in the central and peripheral nervous system, joints, muscles, and in the vestibular, skin, cardiovascular, renal, respiratory or gastrointestinal systems [4]. Some of the most common musculoskeletal system conditions in older adults include neck pain, primary headaches and shoulder pain [5,6]. Neck pain affects all population groups, especially older adults [5]. In fact, almost 75% of the population suffer from neck pain at some point in their lives, and it is the most frequent cause of visits to health professionals, involving a high economic cost [7].

On the other hand, the multifactorial-based alteration of the postural pattern constitutes a change that affects this population, especially women. These alterations derive mainly from the changes caused by the ageing process and a bipedal position due to modifications in the passive and active stabilizing elements of the spine [8]. In addition, other factors such as sarcopenia, osteopenia, reduced levels of physical activity and tendon and ligament weakness also play a role [9]. These alterations can also lead to increased kyphosis of the dorsal spine and anterior displacement of the shoulders, associated with a static alteration of the position of the scapula [10]. This increase in thoracic kyphosis induces a transfer in the centre of gravity and may increase the number of falls [11] and, consequently, the risk of fractures and eventually affect functionality [12].

In accordance with international guidelines on healthy ageing, it is important to highlight the need for strategies aimed at promoting an active lifestyle among the older adult population, with a focus on regular exercise [13]. One of the simplest, most cost-effective and environmentally sustainable ways of performing aerobic exercise is walking. Studies have focused on the physical, cognitive and anti-nociceptive effects of walking in older adults specifically, as aerobic exercise has been highlighted as a valid therapeutic modality for chronic musculoskeletal pain [14]. Walking has been found to lead to subjective improvements in quality of life and pain in older adults [15,16,17,18,19,20].

Physical therapy, on the other hand, includes techniques such as manual therapy, which offers an effective option for relieving neck pain and improving posture [21]. In addition, it can stimulate vasodilation and increase local blood flow, helping to improve range of motion, reduce pain and promote changes at the tissue level [22]. Myofascial release techniques have also shown positive effects for the treatment of chronic shoulder pain [23,24] in fibromyalgia [25], as well as on joint range of motion and pain in non-pathological symptomatic people [26]. According to Cunha and colleagues [27], the combined application of stretching and manual therapy techniques has positive effects on mobility in patients with chronic neck pain. On the other hand, preliminary results show that costotransverse joint mobility and diaphragm release could modulate the autonomic nervous system, reducing the resting heart rate in patients with chronic obstructive pulmonary disease [26].

Accordingly, if all these techniques have been shown to have positive effects on pain, quality of life and functional variables, a simple protocol that includes similar adapted self-administered techniques may also be beneficial. Self-administration of a guided manual therapy model means that patients could apply the protocol on their own or as an aid to other medical treatments. Other authors have conducted studies of self-administered treatment in oncology patients, resulting in symptom relief and improved quality of life [28].

It is worth noting that multicomponent training strategies promote an improvement in the general health condition of older adults [29]. However, we have not found evidence of protocols using self-administered manual therapy alone or combined with high-intensity walking to test the effect that they can produce together on musculoskeletal pain, posture and functionality in older adults. It is also important to highlight that guided exercise, performed independently and supervised by professionals, is an effective strategy for older adults to achieve the exercise levels recommended by international guidelines [30].

The present study hypothesized that self-assisted manual therapy involving soft tissue and cervical-thoracic joint mobilization, combined with high-intensity walking, can reduce musculoskeletal back pain and improve functionality in older adults, not only immediately but also in the medium term, promoting health responsibility and disease prevention in this population. The present study aimed to analyse the effect of a combined protocol of self-assisted manual therapy and high-intensity walking vs. high-intensity walking alone on back musculoskeletal pain, functionality, and posture in older adults. In addition, the aim was also to find out whether the effect is only short-term or whether it may be maintained medium-term.

## 2. Materials and Methods

### 2.1. Study Design

A randomized clinical trial, conducted in a multicentre, double-blind, parallel-group design with an active comparator was conducted in the Laboratories of the Faculty of Physiotherapy, University of Valencia (Spain) and the Faculty of Physical Education and Sport, Charles University (Prague) from October 2021 to May 2022. The potential bias arising from a sample of two culturally and socially different locations was controlled by the stay of some co-authors in the collaborating country. This exchange facilitated the transfer of the methodology for both the evaluation and execution of techniques uniformly. Additionally, monthly follow-up checks were conducted to monitor the consistency and accuracy of the procedure. Participants were randomly divided into two groups: (a) manual therapy and high-intensity walking group (MTWG), who underwent self-assisted manual therapy sessions, plus guided and supervised high-intensity walking; (b) Comparator-controlled group (CG) who performed guided and supervised high-intensity walking. Both interventions lasted 4 weeks with a total of 8 sessions (twice a week). All parts of the protocol were implemented in a group setting and were always guided by an experienced physiotherapist. Study variables were assessed at three different times: at baseline (T0), after the 4-week intervention (T1), and after a follow-up period of 4 weeks following the end of the intervention (T2), where participants were asked not to replicate the intervention.

All participants were fully informed about the aim of the study and experimental procedures and provided written informed and image consent. The study was conducted in accordance with the Declaration of Helsinki [31] and approved by the Ethics Committee for Research on Humans University of Valencia (No. 1393203) and registered as part of a larger study in the ClinicalTrials.gov database (Identifier: NCT04345211). The study was conducted following the guidelines of the Consolidated Standards of Reporting Trials (CONSORT).

### 2.2. Participants

The present study included community-dwelling older adults who met the following inclusion criteria: aged 60–80 years, with non-specific chronic musculoskeletal or osteoarticular pain (>3 months), non-smokers (>6 months), and willing to participate. The following exclusion criteria were considered: history of respiratory or cardiac condition, inability to walk without technical aids or assistance, acute musculoskeletal or osteoarticular pain at the time of assessment, contraindications for manual therapy or exercise, cognitive impairment (Mini-Mental State Examination < 25 points) [32].

### 2.3. Randomization and Blinding

Participants were informed in the informed consent form about their allocation to one of two possible interventions and signed their consent to participate knowing that they could be assigned to either group. Their data were placed in concealed opaque envelopes for random assignments and divided into two blocks corresponding to the intervention groups. To minimize bias in data collection, an independent assessor, blinded to participant allocation, performed the assessments and recorded the data. This assessor also provided the questionnaires and clarified any doubts. To ensure double blinding, a statistician, unaware of the treatment group assignments, analyzed and interpreted the results.

### 2.4. Outcomes

In each of the institutions, a physiotherapist with 15 years of experience in treating musculoskeletal disorders in older adults conducted individual, face-to-face assessments. Before the session, procedures were explained in detail to all participants. An extensive anamnesis covering sociodemographic, anthropometric, and clinical data was conducted, followed by the main study variables. The evaluation period was conducted along the 5 days before the intervention and then along the 5 days after the end of the intervention.

Sociodemographic characteristics collected were age, sex, educational level, marital status, and employment status. The measured anthropometric characteristics were as follows. Body weight was measured using a Tanita BC 601 (TANITA Ltd., Tokyo, Japan); height assessed with a SECA 213 stadiometer (Seca Ltd., Hamburg, Germany); and body mass index (BMI) was calculated (kg/m^2^). Finally, comorbidities were assessed using the abbreviated Charlson Comorbidity Index [33], which encompasses eight medical conditions (cerebrovascular disease, diabetes, chronic obstructive pulmonary disease, heart failure/ischaemic heart disease, dementia, peripheral arterial disease, chronic kidney failure and cancer). The scores range from 0–10, where 0 is no comorbidity, and 10 is high comorbidity; absence of comorbidity is considered between 0 and 1 points, low comorbidity when the score is 2, and high comorbidity when it is ≥3.

#### 2.4.1. Primary Outcomes

- Pressure pain threshold (PPT): Measured by establishing the minimal pressure (kg/cm^2^) at which pain is induced using pressure algometry (Wagner Instruments FDK 20, Greenwich, USA). The patient was seated, and the trapezius muscles were assessed bilaterally, applying pressure 3 cm distal to the midpoint of the upper trapezius muscle, performing three measurements on each muscle, with a thirty-second rest between them. The physiotherapist instructed the patient to say “stop” when their sensation changed from pressure to pain. The average of the three scores was obtained for analysis. ICC = 0.91 (95% CI: 0.82, 0.97) [34].
- Pain Intensity: Participants were asked to rate their overall perception of musculoskeletal back pain measured by the Visual analogue scale (VAS), a unidimensional measure of non-specific perceived pain intensity, which has been widely used in diverse adult populations. The pain VAS is a continuous scale comprised of a horizontal or vertical line, typically 10 cm in length (a range of 0 to 100 is established). In order to score pain intensity, the person marked on the scale “no pain” (score of 0) and “worst imaginable pain”. The participants were asked to report “current” pain intensity or pain intensity “in the last 24 h”. A higher score indicates greater pain intensity [35]. The VAS has a high internal consistency (0.92) [36].

#### 2.4.2. Secondary Outcomes

- Functional capacity, using the five Times Sit-to-Stand Test (5XSST) [37,38]. This test assesses risk of recurrent falls, with the cut off score of >15 s, indicating poor functional capacity and risk of falls [39]. For the 5XSST, participants were asked to stand up from and sit down on a slightly padded 43 cm high armless chair as quickly as possible 5 times. Participants folded their arms across their chest and were instructed to stand up completely and make firm contact when sitting. Timing began on the command “go” and stopped when the patient sat down again after the 5th repetition. Participants were allowed a practice trial of 2 repetitions before the timing of 2 test trials of 5 repetitions. The fastest of the 2 test trials was used in subsequent analysis.
- Change in thorax position, based on the distance between the acromion and base, measuring the linear distance from the posterior edge of the acromion to the flat surface. Participants were requested to lie supine on a standard stretcher and adopt their natural relaxed posture. As described by Sahrmann (2001), the participants rested with their arms alongside their body and with the elbows flexed in contact with the lateral wall of the abdomen [40]. The hands rested gently on the abdomen, placing the glenohumeral joint in slight internal rotation. The investigator measured the linear distance in mm using a standard plastic transparent ruler. Without exerting any downward pressure, the base of the protractor was placed on the stretcher, and the vertical side was placed adjacent to the lateral aspect of the acromion. Each measurement for each side was measured 3 times in succession, and on each occasion, the right angle was replaced as previously described. Furthermore, in the same study, they demonstrated the reliability of the test with an ICC of between 0.92 and 0.93 for people with symptoms and between 0.90 and 0.93 for people without symptoms [41].

### 2.5. Intervention

1. Self-administered manual therapy (MTWG). A self-administered manual therapy protocol was carried out in 30-min sessions, including seven techniques adapted from Yilmaz Yelvar et al., 2016 [42]. In addition, two myofascial techniques were added, making a total of 9 techniques. At the beginning and end of each session, breathing exercises were performed to make the participants aware of their breathing [42]. Subsequently, a battery of 9 specific techniques were self-administered: (1) Neurolymphatic technique [43,44]; (2) Suboccipital decompression technique [42]; (3) Gliding technique on the cervical vertebral joints (anterior/posterior) [42]; (4) Inhibition and stretching of the sternocleidomastoid and trapezius muscles [45]; (5) Gliding technique on the sternoclavicular joint using an anterior/posterior direction [42]; (6) Mobilization of the scapulothoracic joint and gliding of the thoracic vertebral joints [42]; (7) Myofascial release technique of intercostal muscles and paravertebral muscles [42] (Yilmaz Yelvar et al., (2016); (8) Diaphragmatic release technique [42]; (9) Rib elevation technique [42]. As noted, breathing exercises were performed at the beginning and the end of each session [42] (Supplementary S1). In each of the centres the implementation of all techniques was guided by a physiotherapist with more than 20 years of experience in manual therapy.
2. Walking activity. Walking was performed immediately after the manual therapy intervention in the case of the MTWG, as this has been shown to enhance the synergistic effect of combining the two interventions [46]. It was implemented by the same physiotherapist who guided the self-administered manual therapy. A vigorous or high-intensity walking protocol was followed, having a weekly walking goal of 75 min per week of vigorous aerobic activity, consistent with recommendations of the World Health Organization 2010 [47]. This protocol consisted of supervised walking on a 400 m circular terrain. The Borg perceived exertion scale was used to determine exercise intensity during the walking protocol [48]. In this way, walking goals were customized based on each participant’s level of physical fitness. Before starting the walking time in each session, this scale was described to the participants, and the intensity at which they should walk in each session was indicated. The structure of the walking sessions began with a warm-up where participants walked at <12 grades of the Borg scale, with a duration of 10 min. After the warm-up, participants walked at a target intensity of minimum of 16 on the Borg scale for 40 min, reaching an intensity at which they could not hold a conversation [49]. Afterwards, a cool-down was performed for 10 min.

### 2.6. Sample Size Calculation

The sample size was calculated using G*Power 3.1.9.7. A priori power analysis was performed for two independent groups with the aim of detecting small to moderate effect sizes (Cohen’s d = 0.25) with α = 0.05 and 1 − β = 0.95. A Cohen’s d value of d = 0.25 was chosen based on prior studies, which showed that non-pharmaceutical interventions tend to have moderate to small effects on pain reports, as measured by the VAS [50]. The results indicated that a sample size of 44 participants would be required, and considering a loss of 20%, a total of 53 participants were needed.

### 2.7. Statistical Analysis

All statistical analyses were conducted in IBM SPSS Statistics 29. First, Pearson’s bivariate correlations were calculated to determine any association between outcomes. For example, as prior studies show, it is plausible for pain-related variables to covary systematically [51]. A statistically significant correlation between outcomes would justify conducting repeated measures, multivariate analysis of (co)variance, or RM-MANCOVA. Box’s M and Mauchly’s Sphericity tests were conducted to ensure our RM-MANCOVA models were trustworthy. A non-significant *p*-value for this test would indicate that the sphericity assumption has been met. However, if the Sphericity test fails, certain corrections can be applied based on the value of the Epsilon statistic.

Four RM-MANCOVA models were generated to test our hypotheses, two for our primary outcomes (Models A and B), and two for our secondary outcomes (Models C and D). In all models, the dependent variable was measured at three data points, mainly (T0) baseline, (T1) post-intervention, and (T2) follow-up (one month after).

Model A tested for significant differences in estimated means of participants’ pain by PPT. Model B tested for significant differences in estimated means among participant groups in overall pain as captured by the VAS. Model C tested functional differences as measured by the 5XSST. Model D tested for significant differences in participants’ thorax position as measured by distance from the posterior edge of the acromion to the base. For our between-subjects factor, we entered a dummy coded variable, in which the control group was coded as “0”, and the treatment group was coded as “1”. No control variables were entered in any of the four models.

## 3. Results

**Figure 1:**
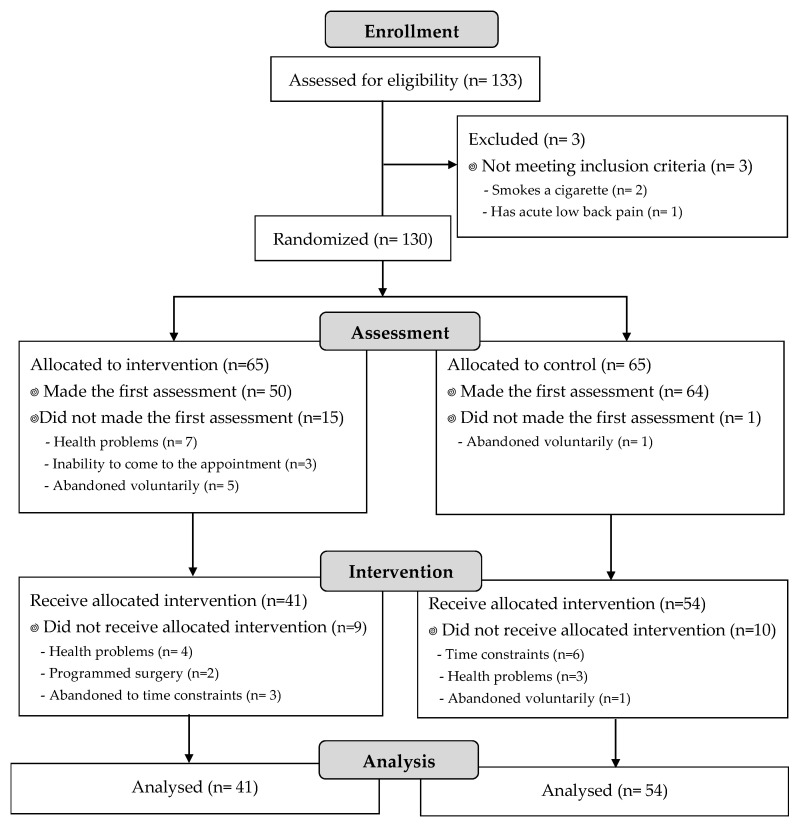
Flow chart according to CONSORT Statement for the Report of Randomized Trial.

From the 133 initial participants, 130 met the inclusion criteria and were allocated to intervention. However, 16 dropped out before the first assessment, so 114 were initially assessed (Table 1). Before intervention, some participants dropped out; therefore, 95 (41 in the MTWG and 54 in CG) completed the study and were analyzed (Figure 1).

The mean age was 69.44 (4.24) years, 56.1% were married, and 65.8% had university studies. The mean BMI was 26.18 (4.24) kg/m^2^ (Table 1).

Table 2 shows that all data points were correlated for each of our primary and secondary outcome measures. This correlation means that the baseline measurement was significantly correlated with the post-test and the follow-up measurements, justifying the use of an RM-MANOVA approach to isolate the effect of those correlations (See Supplementary S2 for a more detailed explanation).

Table 3 shows estimated marginal means across groups for all outcome variables.

### 3.1. Primary Outcomes

Model A. For Model A, the univariate test results for the overall solution revealed significant between-subject (F (1; 94) = 8.55, *p* < 0.01, partial η2 = 0.09; Cohen’s d = 0.62) and within-subject (F (2; 188) = 5.28, *p* < 0.001, partial η2 = 0.07; Cohen’s d = 0.57) differences in estimated marginal means of PPT. More importantly, however, a statistically significant between-within-subjects interaction effect was found (F (2; 188) = 2.97, *p* < 0.05; partial η2 = 0.03; Cohen’s d = 0.36), with the MTWG showing an improvement both at T1 and T2. This means that the CG reported a statistically significant higher pain threshold than the MTWG group in all three datapoints, but the overall trend for the MTWG was significantly different than the trend for CG.

**Figure 2:**
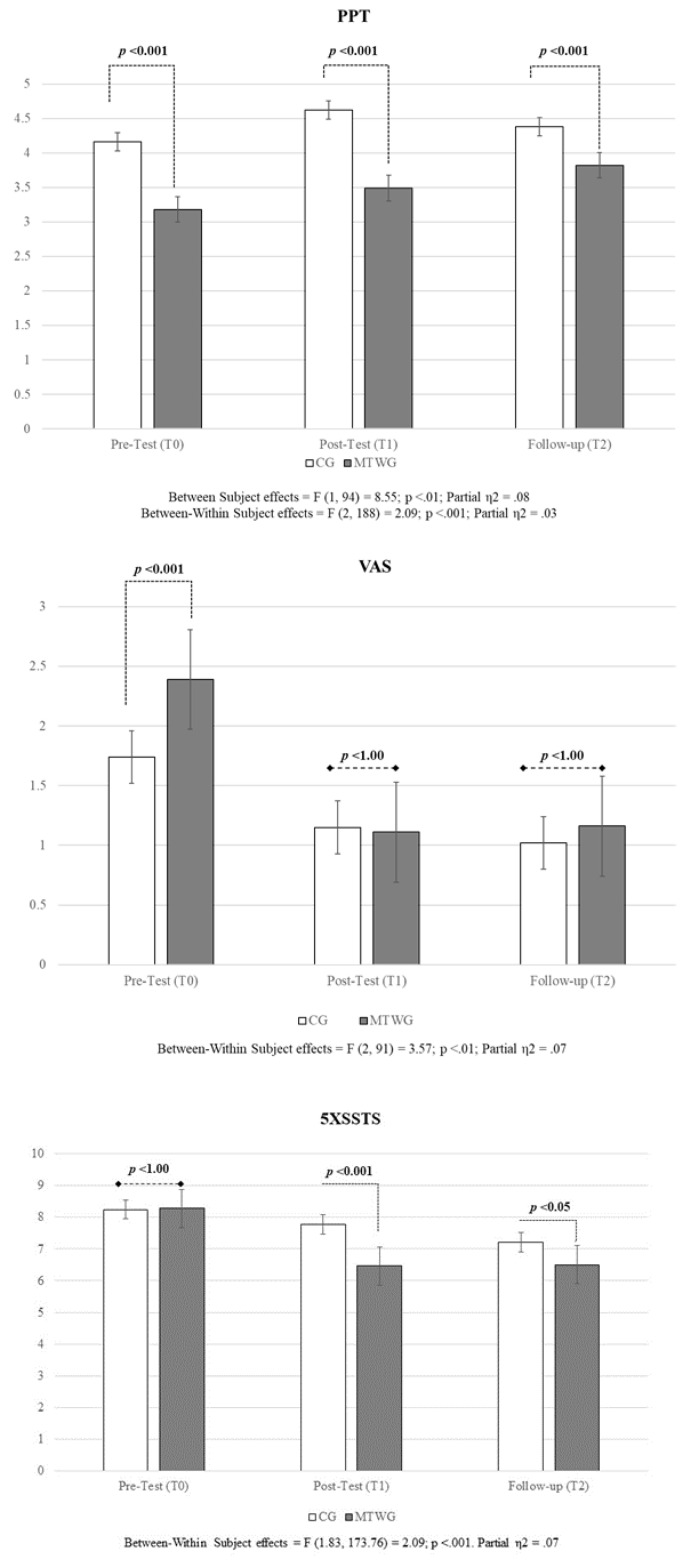
Estimated Marginal Means for self-reported and functional outcomes (PPT, VAS, 5XSST). Note: MTWG: Manual therapy and high-intensity walking group; CG: Comparator-controlled group; PPT: Pressure pain threshold; VAS: Visual analogue scale; 5XSST: five Times Sit to Stand Test.

Model B. No significant between-subject differences were observed (F (1, 92) = 1.02, ns) in Model B. However, and more importantly, the multivariate results revealed statistically significant within-subject differences in estimated marginal means of the VAS (Wilks’ Λ = 0.54, F (2, 91) = 38.91, *p* < 0.001; partial η2 = 0.46; Cohen’s d = 1.85). Similarly, Model B detected significant between-within differences among participants (Wilks’ Λ = 0.93, F (2, 91) = 3.58, *p* < 0.05; partial η2 = 0.07; Cohen’s d = 0.56), with the MTWG reporting less pain at T1 and T2. Figure 2 shows how while the MTWG reported a significantly higher estimated mean VAS score than the CG at the pre-test (T0), this difference disappears at the post-test (T1), and both estimated means remain stable up to one month after the treatment (follow-up, T2).

### 3.2. Secondary Outcomes

Model C. For Model C, no between-subject effects were detected. However, the univariate test results revealed significant within-subject (F (1.83; 173.76) = 31.95, *p* < 0.01, partial η2 = 0.25; Cohen’s d = 1.17) and between-within subject interaction effect of the 5XSST (F (1.83; 173.76) = 6.65, *p* < 0.01, partial η2 = 0.07; Cohen’s d = 0.52), in favor of the MTWG. This means that while the estimated marginal means did not differ among groups in all data points, after the intervention (T1), the MTWG shows lower scores in the 5XSST than the CG, and this change in trend remains stable in the follow-up datapoint (T2). Table 3 shows estimated marginal means (EMMs) for all outcomes, and Figure 2 illustrates EEMs and their respective SE for Models A, B, and C.

**Figure 3:**
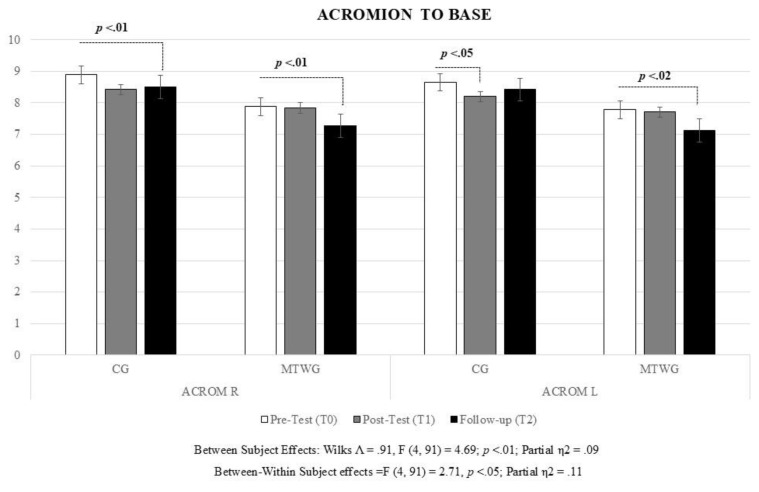
Estimated Marginal Means for distance between acromion to base. Note: MTWG: Manual therapy and high-intensity walking group; CG: Comparator-controlled group; ACROM R: acromion position right; ACROM L: acromion position left.

Model D. The multivariate results for Model D show significant between-subject differences in estimated marginal means (Wilks’ Λ = 0.91, F (2, 93) = 4.69, *p* < 0.01; partial η2 = 0.09; Cohen’s d = 0.64). Further, statistically significant within-subject differences were found (Wilks’ Λ = 0.89, F (4, 91) = 2.69, *p* < 0.05; partial η2 = 0.11; Cohen’s d = 0.69). Furthermore, Model D detected significant between-within subject differences among participants (Wilks’ Λ = 0.89, F (4, 91) = 2.69, *p* < 0.05; partial η2 = 0.11; Cohen’s d = 0.69) for both the right and left side of the acromion position in favor of the MTWG. In other words, the CG report higher scores in the distance between the right acromion to base and the distance between the left acromion to base than the MTWG group, but the overall trend is different for each treatment. Whereas the MTWG shows a decreasing trend (especially between T1 and T2) for both the right and left distance between acromion to base, the CG group shows a decrease between pre (T0) and post-test (T1) but tends to increase in the follow-up test (T2). Figure 3 illustrates the EMMs for Model D.

It is to be noted that the Mauchly’s Sphericity Test was significant for both right acromion position (ACROM R) (W_(2)_ = 0.87; χ2_(2)_ = 12.51; *p* < 0.002) and for left acromion position (ACROM L) (W_(2)_ = 0.86; χ2_(2)_ = 14.30; *p* < 0.001). To ensure the robustness of our findings, we employed the Huynh-Feldt correction for both within and between-within subject tests and inspected the univariate solution. For the distance between the right acromion to base, the within-subject effect was significant (F (1.83, 177.78) = 5.93, *p* < 0.01) and so was the case for the distance between the left acromion to base (F (1.80, 164.54) = 5.20, *p* < 0.01). However, the between-within subject effect was only marginally significant for the distance between the right acromion to base (F (1.83, 177.78) = 2.67, *p* < 0.10) but full significance was achieved for the distance between the left acromion to base (F (1.80, 164.54) = 4.27, *p* < 0.05). Consequently, we suggest caution when interpreting between-within effects of the distance between the right acromion to base.

## 4. Discussion

The results of this study showed that older adults who received the combined program of self-assisted manual therapy and high-intensity walking (MTWG) significantly reduced their pain in the trapezius assessed with algometry, as well as patients’ overall musculoskeletal pain intensity, assessed with the VAS, when compared to the CG. Moreover, such a significant reduction was also maintained in the follow-up assessment. The functional capacity, measured with the 5XSST, of the MTWG significantly improved after the intervention although this situation was not maintained in the follow-up. The acromion position also showed an improvement for those in the MTWG with a moderate effect size. Although we also observed an improvement in the CG variables, such improvements did not reach statistical significance when compared to the MTWG.

Currently, the world’s population is mainly between 15 and 64 years of age (World Bank Open Data, s. f.), with a high proportion of people over 60 years of age. This phenomenon occurs in a global context where international policies not only promote environmental sustainability but also health sustainability. In this sense, it is essential to promote activities and programs that, in addition to being environmentally friendly, are low-cost and can be reproduced independently of the economic resources of each country, thus guaranteeing their accessibility and effectiveness at a global level. These aspects were considered in the design of the present study, which, to the extent of our knowledge, is the first to apply self-assisted manual therapy combined with high-intensity walking on older adults.

In the cervicothoracic region the upper trapezius appears to be the most sensitive of the muscles in both sexes [52], and its improvement can be explained by the applied treatment since there were techniques which were directly applied to the trapezius. A previous study showed that adults with head and neck pain improved their main trigger points of the cervical muscles after a 6-week massage program [53] administered by a therapist. In our study, the manual therapy protocol was effective when applied in an older adult population who have highly prevalent rates of musculoskeletal pain [54], plus it was self-administered.

On the other hand, it should be noted that the results of this work in relation to the perception of pain are similar to those obtained in other studies that have used different manual therapy techniques in patients with neck pain. Thus, Nasir et al. (2021) [55] applied manual therapy techniques (although they did not specify the applied techniques) and a home exercise program in young adults, and both treatment options improved pain and disability due to cervical pain. Arjona Retamal et al. (2021) [56] used suboccipital induction techniques applied manually and by using a tool in young adults to achieve improvements in mobility, pain perception and pain intensity. Nakamaru et al. (2019) [57] carried out a self-mobilization thoracic spine technique tool made with two tennis balls fixed with athletic tape in adults, including some older adults. Still, there was no improvement in neck pain or disability, only immediate improvements in mobility in the cervical spine. Therefore, although it has been shown that manual therapy improves these aspects, previous studies do not have a broader application in the thoracic and cervical region, nor are they self-applied manual therapy techniques, but rather passive ones. Moreover, they were focused on younger adults.

Previously, it has been described that walking can prevent cardiovascular disease [58], reduce the risk of falls [59], improve physical fitness [60], and reduce anxiety and depression in older adults. Moreover, Kocur et al. (2017) [61] achieved a reduction in pressure sensitivity in the trapezius with the application of a 12-week Nordic walking program for office workers. Therefore, the CG who also received the high-intensity supervised walking sessions improved in pain, but not with statistically significant differences compared to the MTWG. Therefore, the MTWG, whose treatment included not only walking but also manual therapy, improved in pain-related variables.

Overall, it is confirmed that our study enhances the idea that following physical exercise protocols and learning self-care techniques based on manual therapy techniques can be an effective tool to promote health and wellbeing, as well as to reduce pain and improve areas of pain impact.

In relation to functionality, it was assessed by 5XSST, and although the results obtained at T1 were not maintained at T2, they still were positive results. It is possible that a manual therapy treatment applied to the lower limbs would have also enhanced the results of this combined group at the follow-up. The improvement at T1 can be explained because the manual therapy treatment applied (focused on the back and thorax) may have influenced the translation into greater functionality and performance during the test. Also, by reducing pain, spasm responses are minimized, and greater coordination between the muscle groups involved in the movement is favored. All of this could have contributed to optimizing the mechanics of movement in this test, and positive results in this test encompass key aspects for independence in daily activities, especially in populations at risk of functional decline. Previously, it has been shown that manual techniques and therapeutic exercises directed at the spine and thorax improve mobility, reduce pain and, therefore, optimize the mechanics of the “sit-to-stand” movement [62]. Recent research has confirmed that treatment in these areas is associated with significant improvements in neuromuscular function and performance in mobility tasks [63] and balance [64], which is essential to maintaining functional independence in daily life.

Finally, regarding thorax position, previous evidence has shown that there are postural changes which frequently occur during the aging process [65]. The MTWG reduced the measured distance between the acromion and bed; thus, shoulders had less anterior displacement when compared to the CG. The explanation can be that by applying manual therapy techniques, there was an effective modulation in relieving soft tissue and probably the range of motion [66]. The manual therapy techniques applied included neuro-lymphatic technique in the pectoral and back, mobilizations in the suboccipital, sternoclavicular, scapulothoracic and thoracic vertebral regions, ribs and diaphragm, and trigger point and massage in the sternocleidomastoid and trapezius, myofascial release of intercostal muscles and paravertebral muscles which have shown to be effective in our study. Also, previous studies have shown that pain and function can improve after applying similar manual therapy techniques [21], and they have also shown to be effective for pain relief in others when applied separately [42,43,44,67,68]. Nitayarak and Charntaraviroj (2021) [69] also reported an improvement in posture and elongation of the pectoralis minor, as well as an increase in the strength of the scapular muscles. However, these studies were applied to younger adults. Considering that older adults usually have osteoarticular stiffness of the rib cage, thus suffering from chest stiffness and marked dorsal kyphosis [70], the self-administered manual therapy program offers an option to improve thorax position. There is evidence that self-management can effectively reduce the pain and disability associated with musculoskeletal conditions [71].

In the present study, it was observed that manual physiotherapy had an effect on walking ability. In this regard, preliminary evidence suggests that certain modalities of manual therapy may induce changes in specific gait parameters [72]. Previous studies have demonstrated that, in patients with chronic low back pain, the application of manual therapy at spinal levels—including spinal mobilization through passive accessory intervertebral movements and passive physiological intervertebral movements—promotes a trend toward gait symmetry. This improvement in kinetic and kinematic parameters may be attributed to enhanced proprioception, changes in muscle activity, and a reduction in pain [73]. To the best of our knowledge, our study is the first to apply a self-assisted manual therapy approach in a simple and reproducible way, which the clinician can teach patients to improve these aspects and probably help them to take part in pain relief when it comes to nonspecific pains that are related to age.

Overall, self-care physical therapy techniques for older adults are crucial for several reasons. On the one hand, it promotes the maintenance of mobility, independence, and quality of life, allowing older adults to perform their daily activities with greater ease and safety. On the other hand, exercise improves balance and coordination, significantly reducing the risk of falls and injuries [74]. Moreover, self-treatment promotes autonomy and self-efficacy, as older adults feel more capable and confident in managing their own health. Therefore, implementing a group or individual self-care bodywork program not only improves the physical health of older adults but also promotes their emotional and mental well-being, contributing to a more active and independent life [75].

The importance of a patient being able to self-treat with techniques or actively participate in their pain management lies in several key factors. First, self-care promotes patient autonomy by helping them better understand their condition and take complementary measures to alleviate pain alongside professional treatment. This active participation enhances health literacy, fosters healthy habits, and improves adherence to therapeutic interventions. Additionally, incorporating self-massage techniques and other self-management methods can reduce stress and muscle tension, improve circulation, and create an overall sense of wellbeing, which positively impacts quality of life. Patient involvement in self-care is associated with a reduction in pain perception and improvements in physical and emotional functioning—critical components for the comprehensive management of chronic conditions [76].

In the present study, interventions were supervised by a physical therapist. In order to ensure older adults follow a self-treatment program at home without constant supervision, education and training strategies can be used, providing clear information about the exercises and their benefits. Techniques should be adapted to the mobility and abilities of older adults [77]. Assistive technology, such as mobile apps and tracking devices, can facilitate adherence and supervision by providing instructions and monitoring progress. Encouraging autonomy and recognizing achievements can increase motivation. Implementing these strategies can help older adults carry out the protocols effectively and safely, promoting health and well-being. These aspects should be considered in future studies.

### Strength and Limitations

The strength of the present study lies in the option of a simple yet effective protocol that can be guided by a clinician and self-administered by the patient. Furthermore, it addresses a very common ailment of aging in older adults. The results appear to be robust, as our sample size was substantially larger than the minimum required amount to detect the expected effect sizes. This large sample size is due to the excellent interest that the recruitment phase elicited in the target population.

However, our work is not without limitations. One limitation of the present study is that we did not assess participants’ adherence to the treatment. However, as our treatment is a self-administered practice, it is likely for patients to participate more in their improvement process, as discussed above. Another limitation is that most of the sample were women, and although this is characteristically related to aging populations, greater equality between the sexes would be important in future research. In this regard, a higher participation of women in health-related studies is usually more frequent than their male counterparts, given women’s overall interest in health and wellbeing [35].

## 5. Conclusions

A combined protocol of self-assisted manual therapy and high-intensity walking compared with only high-intensity walking improved the variables in this study, including back musculoskeletal pain, perception of pain, functionality, and posture in older adults in the short term, after 4 weeks of intervention. Additionally, the improvement of pain was evident in the short term and sustained at follow-up, 4 weeks after the end of the intervention.

## Figures and Tables

**Table 1:** Sociodemographic and anthropometric characteristics of the participants.

	Categories/Units	Total Sample N = 114	MTWG N = 50	CG N = 64	*p*
Sex n (%)	Women	85(74.60%)	36 (72%)	49 (76.60%)	0.58 ^B^
	Men	29 (25.40%)	14 (28%)	15 (23.40%)	
Age (mean/SD)	Years	69.40 (4.20)	69.70 (4.30)	69.30 (5.50)	0.65 ^B^
Marital status n (%)	Single	11 (9.60%)	7 (14%)	4 (6.30%)	0.00 ^A^*
	Married	64 (56.10%)	24 (48%)	40 (62.50%)	
	Widowed	20 (17.50%)	9 (18%)	11 (17.20%)	
	Divorced	15 (13.20%)	9 (18%)	6 (9.40%)	
	Separated	2 (1.80%)		2 (3.10%)	
	Other	2 (1.80%)	1 (2%)	1 (1.60%)	
Level of education n (%)	Primary	1 (0.90%)		1 (1.60%)	0.00 ^A^*
	Secondary	38 (33.40%)	16 (32%)	22 (34.40%)	
	University	75 (65.80%)	34 (68%)	41 (64.10%)	
Employment status n (%)	Employed	10 (8.80%)	1(2%)	9 (14.10%)	0.00 ^A^*
	Retired	104 (91.20%)	49 (98%)	55 (85.90%)	
Weight mean (SD)	kg	70.01 (12.45)	69.72 (11.45)	70.23 (13.26)	0.81 ^A^
Height mean (SD)	m	1.63 (.08)	1.63 (0.09)	1.63 (0.79)	0.99 ^A^
Body mass index mean (SD)	kg/m^2^	26.18 (4.24)	26.09 (4.11)	26.25 (4.37)	0.85 ^B^
Charlson Comorbidity index (IRQ)	Total score	0.00 (3.00)	0.00 (2.00)	0.00 (3.00)	0.66 ^C^
Mini-mental Cognitive Test (IRQ)	Scores	34(6.00)	34(3.00)	34(2.00)	0.19 ^C^

MTWG: Manual therapy and high-intensity walking group; CG: Comparator-controlled group; SD: Standard deviation; IRQ: Interquartile range; ^A^: T-student; ^B^: Chi-square; ^C^: U de Mann-Whitney; *: *p* < 0.05 between MTWG vs. CG.

**Table 2:** Means, SD and Pearson’s bivariate correlations.

−	M	SD	1.	2.	3.	4.	5.	6.	7.	8.	9.	10.	11.	12.	13.	14.	15.
1. Group	1.45	0.50	-														
2. PPT–T0	3.64	1.53	−0.29 **	-													
3. PPT–T1	4.17	1.78	−0.31 **	0.72 **	-												
4. PPT–T2	4.12	1.75	−0.17	0.75 **	0.76 **	-											
5. VAS–T0	2.09	1.63	0.24 *	−0.04	−0.08	−0.04	-										
6. VAS–T1	1.10	1.17	−0.01	−0.03	−0.21 *	−0.08	0.49 **	-									
7. VAS–T2	1.10	1.39	0.06	−0.06	−0.10	−0.04	0.72 **	0.56 **	-								
8. ACROM R– T0	8.36	1.90	−0.32 **	0.32 **	0.31 **	0.23 *	−0.15	0.10	−0.07	-							
9. ACROM R–T1	8.13	1.60	−0.20 *	0.20 *	0.23 *	0.19	−0.22 *	0.09	−0.07	0.69 **	-						
10. ACROM R–T2	7.94	1.76	−0.36 **	0.37 **	0.38 **	0.22 *	−0.17	0.08	−0.01	0.62 **	0.75 **	-					
11. ACROM L–T0	8.18	1.84	−0.30 **	0.35 **	0.35 **	0.30 **	−0.15	0.09	−0.06	0.87 **	0.64 **	0.61 **	-				
12. ACROM L–T1	7.99	1.63	−0.18	0.29 **	0.27 **	0.24 *	−0.21 *	0.10	−0.06	0.67 **	0.86 **	0.71 **	0.77 **	-			
13. ACROM L–T2	7.82	1.83	−0.36 **	0.40 **	0.45 **	0.20 *	−0.21 *	0.02	−0.01	0.57 **	0.67 **	0.92 **	0.64 **	−0.01	-		
14. 5XSST–T0	8.39	2.43	0.03	−0.002	0.04	0.16	0.35 **	0.13	0.32 **	−0.16	−0.11	−0.05	−0.07	0.25 *	0.66 **	-	
15. 5XSST–T1	7.26	2.42	−0.29 **	0.06	0.15	0.21 *	0.02	0.21 *	0.27 **	0.10	0.14	0.20 *	0.17	0.13	0.60 **	0.79 **	-
16. 5XSST–T2	6.94	2.34	−0.13	0.08	0.04	0.03	0.15	0.23 *	0.42 **	−0.04	−0.05	0.09	0.02	0.00	0.13	0.60 **	0.79 **

Note: * *p* < 0.05; ** *p* < 0.01; PPT: Pressure pain threshold; VAS: Visual analogue scale; ACROM R: acromion position right; ACROM L: acromion position left; 5XSST: five Times Sit to Stand Test, T0: baseline; T1: after the 4-week intervention; T2: after a follow-up period of 4 weeks following the end of the intervention. Each variable is shown in a row with a number (1., 2., 3., 4., and so forth). The same variable is shown in a column with the same number. The diagonal is blank (“-“) because the correlation of a variable with itself always equals “1”. Below the diagonal, each cell corresponds to a correlation.

**Table 3:** Estimated marginal means (EMM), Standard errors (SE) and 95% CI for self-reported and functional outcomes.

		EMM (SE); [95 CI%]			F-Test
		T0	T1	T2	
PPT	CG	4.16 (0.20); [3.76, 4.57]	4.62 (0.23); [4.17, 5.07]	4.38 (0.24); [3.91, 4.85]	W-S: F (2, 188) = 7.51; *p* < 0.001; Partial η2 = 0.07
	MTWG	3.18 (0.23); [2.72, 3.69]	3.49 (0.25); [2.91, 3.99]	3.82 (0.27); [3.29, 4.36]	
	*F*-Test	B-S: F (1, 94) = 8.55; *p* < 0.01; Partial η2 = 0.08			B-W: F (2, 188) = 2.09; *p* < 0.001; Partial η2 = 0.03
VAS	CG	1.74 (0.21); [1.34, 2.16]	1.15 (0.16); [0.83, 1.48]	1.02 (0.19); [0.65, 1.40]	W-S: Wilks Λ = 0.54, F (2, 91) = 38.91; *p* < 0.0001; Partial η2 = 0.46
	MTWG	2.39 (0.24); [1.91, 2.88]	1.11 (0.19); [0.74, 1.49]	1.16 (0.22); [0.72, 1.60]	
	*F*-Test	B-S: F (1, 92) = 1.02, ns; Partial η2 = 0.01			W-B: Wilks Λ = 0.93, F (2, 91) = 3.57; *p* < 0.01; Partial η2 = 0.07
5XSST	CG	8.24 (0.32); [7.61, 8.87]	7.77 (0.31); [7.16, 8.39]	7.21 (0.31); [6.59, 7.84]	W-S: F (1.83, 173.76) = 7.51; *p* < 0.001; Partial η2 = 0.25
	MTWG	8.28 (0.36); [7.56, 9.00]	6.46 (0.36); [5.75, 7.16]	6.50 (0.36); [5.79, 7.22]	
	*F*-Test	B-S: F (1, 95) = 2.43, ns; Partial η2 = 0.03			B-W: F (1.83, 173.76) = 2.09; *p* < 0.001; Partial η2 = 0.07
ACROM R	CG	8.89 (0.25); [8.39, 9.39]	8.42 (0.22); [7.99, 8.85]	8.50 (0.23); [8.06, 8.95]	W-S: F (1.83, 171.78) = 5.93, *p* < 0.01; Partial η2 = 0.06
	MTWG	7.88 (0.28); [7.31, 8.43]	7.84 (0.25); [7.35, 8.33]	7.27 (0.28); [6.76, 7.77]	
ACROM L	CG	8.65 (0.23); [8.18, 9.11]	8.20 (0.23); [7.75, 8.65]	8.42 (0.23); [7.95, 8.88]	W-S: F (1.80, 169.19) = 5.20, *p* < 0.01; Partial η2 = 0.05
	MTWG	7.78 (0.27); [7.24, 8.30]	7.71 (0.26); [7.20, 8.22]	7.13 (0.26); [6.60, 7.65]	
	*F*-Test	B-S: Wilks Λ = 0.91, F (4, 91) = 4.69; *p* < 0.01; Partial η2 = 0.09			W-B: Wilks Λ = 0.89, F (4, 91) = 2.71, *p* < 0.05; Partial η2 = 0.11

Note: MTWG: Manual therapy and high-intensity walking group; CG: Comparator-controlled group; B-S = Between Subjects; W-S: Within-Subjects; B-W = Between-Within Subjects. PPT: Pressure pain threshold; VAS: Visual analogue scale; 5XSST: five Times Sit to Stand Test; ACROM R: acromion position right; ACROM L: acromion position left; T0: baseline; T1: after the 4-week intervention; T2: after a follow-up period of 4 weeks following the end of the intervention; EMMs = Estimated Marginal Means; CI = Confidence Interval; ns: non-significant.

## Data Availability

Data are available upon reasonable request.

## Supplementary Materials

The following supporting information can be downloaded at: https://www.mdpi.com/article/10.3390/life15060844/s1. Supplementary S1: Self-Assisted Manual Therapy Protocol; Supplementary S2: Analytical Strategy and Robustness Checks.

## Abbreviations

The following abbreviations are used in this manuscript:

MTWG	manual therapy plus walking at high-intensity Group
CG	Comparator-controlled group
PPT	pressure pain threshold
VAS	visual analogue scale
BMI	body mass index
5XSST	five Times Sit-to-Stand Test
ACROM	distance between the acromion and base
