# Supplementary material for: Effects of Self-Assisted Manual Therapy Combined with a High-Intensity Walking Program on Musculoskeletal Pain, Functionality, and Posture in Older Adults: A Multicentre Randomized Controlled Trial
Source: Life (Basel). 2025 May 23;15(6):844. doi: 10.3390/life15060844 (PMC12194483; doi:10.3390/life15060844)
Supplement: Supplementary file 1 [file life-15-00844-s001.zip › Supplementary Material S1.pdf]

SUPPLEMENTARY MATERIAL S1	
**SELF-ASSISTED MANUAL THERAPY PROTOCOL**	
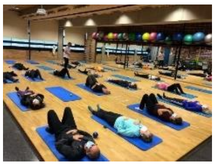	**Respiratory awareness of the abdominal and thoracic area:** The person is placed in supine position and performs thoracic and abdominal respiratory cycles for 5 minutes.
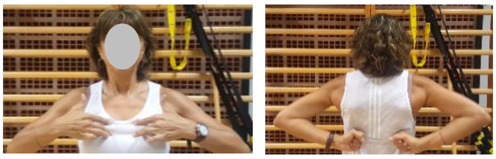	**1- Neurolymphatic technique:** The person performs quick, gentle rubbings with the fingers in the sterno-costal spaces of the 4 upper ribs. Then, the person rubs with the knuckles the posterior area (last ribs and lower lumbar area), rubbing from the thoracic transverse processes (from where the person can reach) to the lumbar transverse processes. They were asked to perform conscious breathing and apply direct, firm, rotary pressure, for 1 minute at each level. This technique is performed for approximately 3 minutes.
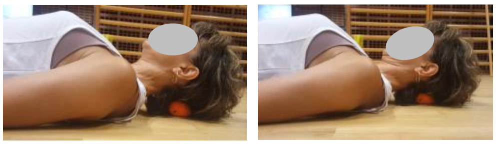	**2- Suboccipital decompression technique:** The person remains in supine position and a small soft ball is placed on the occipital area. The person inhales in neutral position and during the exhalation lowers the chin to the chest, pushes against the ball (suboccipital flexion) and holds the position by lightly pressing the ball for a few seconds. The movement is repeated for 2 minutes.
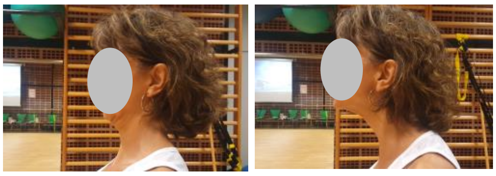	**3- Gliding of the cervical vertebral joints (anterior/posterior):** In standing or sitting position, with the body aligned and practicing conscious breathing, the person moves the head backwards and forwards, inhaling when moving forwards and exhaling when moving backwards. The movement is performed for 2 minutes.
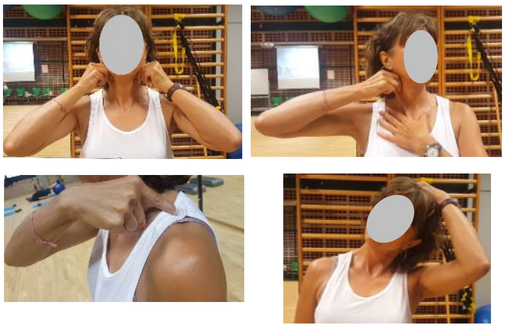	**4- Inhibition and stretching technique of the sternocleidomastoid muscle and trapezius:** The person is standing or sitting and performs pressure on the sensitive trigger points of the sternocleidomastoid muscle for 90 seconds or when the pain disappears and then stretches it. In the same way, the inhibition and subsequent stretching of the trapezius sensitive trigger points is performed. This technique lasts 3 minutes.
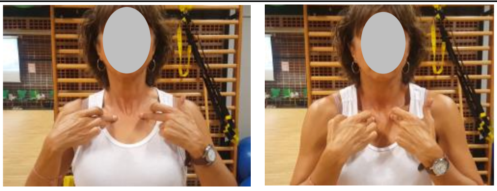	**5- Gliding of sternoclavicular joint (anterior/posterior direction) technique:** The person stays in standing or sitting position with the fingers of both hands on the sternoclavicular joints and performs shoulder movements backwards inhaling and forward exhaling. This technique has a duration of 1 minute.
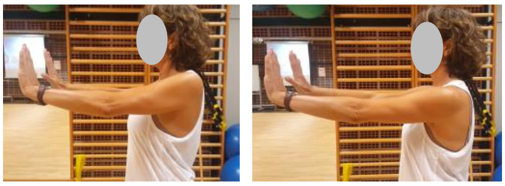	**6- Scapulothoracic joint mobilization and sliding of the thoracic vertebral joints:** The person is standing or sitting with shoulder at 90 degrees of flexion and performs a protraction/retraction movement of the shoulders (anterior/posterior direction). During inhalation the person places shoulders backwards and tries to bring both scapulae together, and during exhalation separates them and kyphosizes the thoracic column. This technique lasts 2 minutes.

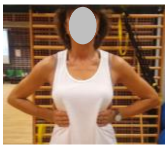 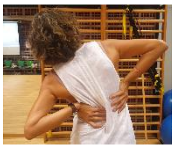 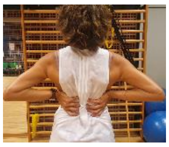 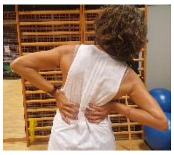	**7- Myofascial release of intercostal muscles and paravertebral muscles:** The person, in standing position, performs rib mobilization (anterior, posterior, lateral). Placing the hands on the ribs (in the anterior thoracic part and then, in the posterior thoracic part), inhalation is accompanied by rib opening and, in exhalation by rib closure, combined with lateralization. The technique is performed for 3 minutes.
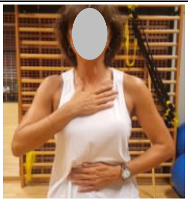	**8- Diaphragmatic release technique:** The person stays in a standing position, with the body well aligned, and places one hand on the thorax and the other on the abdomen and practices conscious abdominal breathing for 2 minutes.
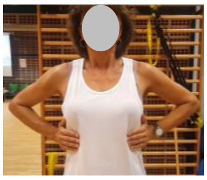	**9- Rib elevation technique:** The subject stays in standing position, with the body well aligned, places the hands on the sides of the thorax on the ribs. The person inhales to assist the rib opening and relaxes during the exhalation. This technique is performed for 2 minutes.
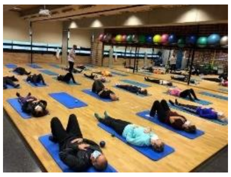	**Abdominal and thoracic respiratory awareness:** The person stays in supine position and performs thoracic and abdominal respiratory cycles for 5 minutes.
