# Supplementary material for: Effects of Self-Assisted Manual Therapy Combined with a High-Intensity Walking Program on Musculoskeletal Pain, Functionality, and Posture in Older Adults: A Multicentre Randomized Controlled Trial
Source: Life (Basel). 2025 May 23;15(6):844. doi: 10.3390/life15060844 (PMC12194483; doi:10.3390/life15060844)
Supplement: Supplementary file 1 [file life-15-00844-s001.zip › Supplementary Material S2.pdf]

Supplementary material S2. Analytical Strategy and Robustness Checks.

Data Analysis

All statistical analyses were conducted in IBM SPSS Statistics 29. First, Pearson's r bivariate correlations were calculated. As prior studies suggest, it is plausible for pain-related variables to covary systematically [51]. A statistically significant correlation between pain measures would justify conducting a repeated-measures, multivariate analysis of (co)variance, or RM-MANOVA.

General linear models, such as an RM-MANOVA, have substantive advantages over linear regression models when testing the efficacy of interventions. For example, the correlation between dependent variables and time effects can be corrected with an RM-MANCOVA. This approach allows for testing the statistical significance of between-subject and within-subject effects, as well as their possible interaction effect (between-within interaction effect). Further, when a general linear model proves to be trustworthy, corrected marginal means for each group can be easily derived from the model (i.e., estimated marginal means, or EMM). EMMs for each group were calculated, and Bonferroni's correction when conducting pairwise comparisons was applied.

Four RM-MANOVA models were generated to test our hypotheses. Models A, B, C and D. For Model A, the dependent variable was an aggregated PPT score, with one value for each of the three temporal data points measured. For our between-subjects factor, we entered a dummy coded variable, in which the control group was coded as "0," and the treatment group was coded as "1." The three data points were (1) baseline, (2) post-intervention, and (3) follow-up (one month after). In Model B, the dependent variable was an aggregated VAS score, which was used as a dependent variable. Again, each metric was assessed at the same three temporal data points as specified in Model A: (1) baseline, (2) post-intervention, and (3) follow-up. A third RM-MANCOVA model, Model C, was specified to test our hypotheses concerning changes in participants' functional capabilities. The dependent variable for Model C was 5XSST, also measured in three data points: (1) baseline, (2) post-intervention, (3) and follow-up. Finally, in Model D, APOSR and APOSL were used as dependent variables. Again, each metric was assessed at the same three temporal data points as specified in Models A and B: (1) baseline, (2) post-intervention, and (3) follow-up. No control variables were entered into Models A, B, C, and D.

Box's M and Mauchly's Sphericity tests were conducted to ensure our RM-MANOVA models were trustworthy. More precisely, Box's M is a statistic that tests the null hypothesis that the covariance matrices in the multivariate solution are equal across groups (i.e., Homogeneity of Variance assumption). A non-significant p-value would indicate that this assumption has been met. Similarly, Mauchly's Test of Sphericity follows the same logic for the within-subject aspect of an RM-MANOVA. Again, a non-significant p-value for this test would indicate that the sphericity assumption has been met. However, if the Sphericity test fails, certain corrections can be applied based on the level of the Epsilon statistic. More precisely, the Greenhouse-Geisser correction should be applied if epsilon < .75. Instead, if epsilon > .75, the Huynh-Feldt correction should be applied to the degrees of freedom in subsequent univariate F-tests.

The results of an RM-MANCOVA can be interpreted as follows. First, the trustworthiness of the overall model must be determined using the tests mentioned above (Box's M and Mauchly's W statistics). If the multivariate solution is deemed untrustworthy, then univariate tests should be preferred. When interpreting the results of a multivariate solution, if the p-value of the Wilks' Lamba

statistic for a given predictor is statistically significant at $p < .05$ in the multivariate solution, that would mean that said predictor is associated with all dependent variables.

Instead, in the univariate solution, the effect of a predictor on a given dependent variable is determined by an F-test. If the sphericity assumption is not met, the F-test can be adjusted employing corrected degrees of freedom if necessary (employing the Greenhouse-Geisser or the Huynh-Feldt corrections). If the significant test of the (corrected) F-value is significant at the $p < .05$ level, that result would support an association between the predictor and each dependent variable.

Lastly, given that an RM-MANCOVA analysis reports Partial Eta squared (partial η^2) as an indicator of effect size rather than Cohen's d, any partial η^2 scores must be transformed into Cohen's d effect sizes to ease its interpretation. According to Cohen's method, the magnitude of the effect is classified as small (0.20–0.49), moderate (0.50–0.79), or large (> 0.80).

Robustness checks

Correlations between outcome measures

Table 2 shows Means, Standard deviations (SD) and our study variables. Pearson's bivariate correlations are shown in the lower diagonal of Table 2. Our results show that, as we expected, PPT-T0 was significantly and strongly correlated with PPT-T1 ($r = .72$, $p <.001$) and PPT-T2 ($r = .75$, $p <.001$). In turn, PPT-T1 was strongly correlated with PPT-T2 ($r = .76$, $p <.001$). Similarly, VAS-T0 was significantly and moderately correlated with VAS-T1 ($r = .49$, $p <.001$) and strongly correlated with VAS-T2 ($r = .75$, $p <.001$). Furthermore, VAS-T1 was strongly correlated with VAS-T2 ($r = .72$, $p <.001$). Finally, our results also show that 5XSST–T0 was significantly and strongly correlated with 5XSST–T1 ($r = .66$, $p <.001$) and 5XSST–T2 ($r = .60$, $p <.001$). In turn, 5XSST – T1 was strongly correlated with 5XSST–T2 ($r = .79$, $p <.001$).

Table X2 also shows that APOSR-T0 was significantly correlated with APOSR-T1 ($r = .69$, $p < .01$) and APOSR-T2 ($r = .62$, $p< .01$). Similarly, APOSR-T1 was significantly correlated to APOSR-T2 ($r = .75$, $p< .01$). Also as expected, APOSL-T0 was significantly correlated with APOSL-T1 ($r = .77$, $p < .01$) and APOSL-T2 ($r = .64$, $p< .01$). Similarly, APOSL-T1 was significantly correlated to APOSL-T2 ($r = .71$, $p< .01$). When taken as a whole, these correlations justify the use of an RM-MANOVA approach.

Check of assumptions for general linear models

Model A. Box's M test for Model A was significant ($M = 25.76$; $F(6, 54{,}276.26) = 4.14$, $p <.001$), suggesting that the multivariate solution is untrustworthy and univariate tests should be preferred. However, Mauchly's Sphericity test was non-significant ($W_{(2)} = 1.00$; $p < .98$). This result indicates that the sphericity assumption was met. Therefore, Sphericity can be assumed when interpreting univariate analyses.

Model B. Box's M test for Model B was non-significant ($M = 11.15$; $F(6, 48{,}744.56) = 1.79$, $p <.10$), suggesting that the multivariate solution is trustworthy. Similarly, Mauchly's Sphericity test was non-significant ($W_{(2)} = .94$; $\chi2_{(2)} = 5.47$; $p < .07$). This result indicates that both the homogeneity of variance and the sphericity assumption were met. Therefore, the multivariate solution was preferred for model B.

Model C. A detailed inspection of the results of Box's M (M = 24.61; F (6, 54,169.56) = 3.96, $p < .001$) and Mauchly's Sphericity tests ($W_{(2)} = .88$; $\chi 2_{(2)} = 12.51$; $p < .002$) revealed that the multivariate solution was again untrustworthy. Therefore, a univariate approach was adopted. Furthermore, because the epsilon statistic was Epsilon = .92, all degrees of freedom employed in the univariate F-tests were corrected using the Huynh-Feldt correction.

Model D. Finally, a detailed inspection of the results of Box's M test for Model D revealed that the multivariate solution was trustworthy (M = 28.66; F (21, 28,510.14) = 1.27, $p < .18$). However, Mauchly's W statistic was significant for APOSLD ($W_{(2)} = .87$; $\chi 2_{(2)} = 12.51$; $p < .002$) and APOSL ($W_{(2)} = .86$; $\chi 2_{(2)} = 14.30$; $p < .001$) suggesting that the correct univariate solution should be preferred when testing within-subject differences.
